# Developing evidence-based, values-driven skills: WHO-ISPED European public health leadership course 2023

**DOI:** 10.1093/eurpub/ckae189

**Published:** 2025-03-25

**Authors:** Geneviève Chêne, Mili Spahic, Joao Breda, Katarzyna Czabanowska

**Affiliations:** University of Bordeaux, Institut de Santé Publique d’Epidémiologie et de Développement (ISPED/Bordeaux School of Public Health), Bordeaux, France; Inserm, Bordeaux Population Health Research Center, CHU Bordeaux, Bordeaux, France; University of Bordeaux, Institut de Santé Publique d’Epidémiologie et de Développement (ISPED/Bordeaux School of Public Health), Bordeaux, France; WHO Office on Quality of Care and Patient Safety, Athens, Greece; Department of International Health, Care and Public Health Research Institute (CAPHRI), Maastricht University, Maastricht, The Netherlands

Effective leadership is the ability to share a vision and take decisive action grounded in values and human rights. This is essential for navigating the complex and challenging landscape of public health today, and forging a clear, determined route forward. This capacity has been significantly tested for public health leaders at all levels, from those in top leadership positions to mid-level roles and beyond, as they work together across organizations to respond durably and efficiently to the Coronavirus Disease (COVID)-19 pandemic. Even in the face of uncertainty, these leaders have worked together to safeguard public health.

The requirements and expectations for public health leaders, like those for leaders in other fields, are extremely high. They must not only navigate volatility and uncertainty but also manage many paradoxes [1]. This demands highly strategic leaders with a broad vision, equally skilled at execution and capable of making rapid, concrete decisions to pave the way toward the future. Additionally, every leader must demonstrate the humility to be an active listener, acknowledging the diversity of those who may possess more relevant expertise and insights, while making bold decisions. Leaders should also be willing to make compromises and remain flexible in their approach, while maintaining trust and integrity as the foundation of all their actions, with a high degree of empathy, kindness, and authenticity. These traits are essential for fostering co-ownership of the transformation process. Effective alliance-based leadership, collaboration with authorities and stakeholders at all levels of society, and communication with the public, are shaped by these factors.

Training in public health leadership is therefore critical for strengthening the next generation of public health professionals aspiring to be leaders, ensuring stronger and more effective leadership to address complex challenges in public health and improve outcomes with the required agility. How can public health leaders be best developed and empowered to make evidence-based decisions for health benefit?

In 2022, supported by the strategic and direction-setting WHO European Programme of Work, 2020–25—“United Action for Better Health in Europe” and the Regional Director’s vision to leave no one behind, WHO Europe partnered with the Italian National Institute of Health to launch the 1st WHO European Public Health Leadership Course (EPHLC), the first course of its kind, in Rome [2].

This inaugural session focused on the significance of cultivating leaders’ emotional intelligence and organizational resilience skills for effective leadership in the face of persistent and complex challenges, including multiple simultaneous emerging crises. Resilience emphasizes the ability to adapt, bounce back from challenges, and maintain mental well-being amidst adversity. These skills contribute to building strong relationships, fostering collaboration, managing workforce stress and burnout, and restoring a forward-thinking mindset. Involving diverse stakeholders and perspectives in interdisciplinary teams is also essential for the resilience of organizations.

In 2023, building on the success of the 2022 session in Rome, WHO Europe partnered with the University of Bordeaux School of Public Health (ISPED), the Graduate Program on Digital Public Health, the Inserm Bordeaux Population Health Research Center, and the Care and Public Health Research Institute (CAPHRI) of the University of Maastricht to organize the 2nd WHO European Public Health Leadership Course. This course continued to promote high standards in public health leadership practice, aligned with the WHO-ASPHER roadmap to professionalization [3].

The 2023 course was designed to strengthen future leaders by gaining insight into one’s values and responsibilities, developing core competencies, enhancing evidence-based decision-making, and fostering emotional resilience. The program included lectures, panel discussions, and problem-based learning (PBL) sessions on key public health topics such as health systems, policies, and digital innovations. It also focused on social inequities, ethical leadership, crisis management, and effective communication, while incorporating personal development activities like workplace conflict resolution and team bonding.

Building on this foundation, the course aligned with a comprehensive reference framework [4], covering essential leadership competencies such as systems thinking, political and collaborative leadership, communication, emotional intelligence, organizational learning, and ethics. By equipping participants with these skills, the program prepared them to navigate complex systems and drive meaningful transformations in public health. Special emphasis was placed on ethical professionalism, values-based decision-making, and empowering leaders to incorporate social justice principles, embrace diversity, and tackle ethical dilemmas.

In an era of information overload, the training highlighted evidence-based decision-making as a fundamental leadership skill, enabling leaders to engage with research and improve public health outcomes. Through transformative leadership [5], experiential learning and mentoring, the course addressed leadership in various contexts (self, team, other leaders, systems, and organizations), while using concrete PBL exercises [6] to tackle significant public health challenges, such as access to primary health care, digitization and artificial intelligence, prevention of arterial hypertension, misinformation, and health equity. This connection of leadership competencies with real-world issues served as a guiding thread throughout the program, fostering an integrated and impactful view to public health leadership.

This program also fostered a network of practices among participants through collaborative learning, expert connections, and continued collaboration. Through PBL sessions and group activities, participants worked together to solve real-world public health challenges, encouraging collaboration and the sharing of diverse solutions. Additionally, the program featured lectures and discussions with leading public health professionals, offering the 53 participants opportunities to engage with experts and form relationships that could continue beyond the course. Furthermore, by addressing shared challenges in public health, trust was built among participants, laying the groundwork for future collaboration on initiatives, research, or policy development within an established active network of peers.

Participants rated various aspects of the program on a scale from 0 (not satisfied) to 5 (very satisfied), assessing program content quality, educational sequence, balance between lectures and interactive exercises, organizational support, and overall satisfaction. Additionally, participants provided open-ended feedback for areas of improvement on aspects like the hybrid format (online and in-person), or content coverage. Of the 35 respondents, 27 were “very satisfied” and 8 were “satisfied.” Twenty-three participants awarded the highest rating to program content, while 12 rated it 4 out of 5. The balance between lectures and interactive exercises and the hybrid structure (online in week 1, in-person in week 2) also received high ratings (4 or 5 from 34 participants). Areas for improvement included enhancing group work during the online week to foster cohesion ahead of in-person sessions and increasing feedback on PBL sessions to support more diverse group interaction. Recommendations for future courses highlighted the inclusion of more on debate and media communication skills, negotiation, multiculturalism, climate change, and misinformation management. Finally, feedback praised the course organization, engaging social activities, and relevant instructors but suggested more rest time and a greater geographic diversity of participants. Improvements that will certainly be addressed for the 2024 session.

As the field evolves, so will the competencies required of its leaders. The course aims to enhance responses to health risks, threats and damages, and bridge the gap between knowledge and action, preparing the next generation of public health leaders to address complex challenges and contribute to a European public health innovation network, ultimately positively impacting population health outcomes.

## Data Availability

The data used in this article are available from the corresponding author upon reasonable request.
